# Genetic and immune changes in Tibetan high-altitude populations contribute to biological adaptation to hypoxia

**DOI:** 10.1265/ehpm.22-00040

**Published:** 2022-10-15

**Authors:** Jun Bai, Lijuan Li, Yanhong Li, Liansheng Zhang

**Affiliations:** 1 Institute of Hematology, Lanzhou University Second Hospital, Lanzhou 730000, China; 2 Gansu Key Laboratory of Hematology, Lanzhou 730000, China; 3 Dingxi People’s Hospital, Dingxi 730500, China

**Keywords:** High altitude, Tibetan, Immune, Genetics, Hypoxia

## Abstract

**Background:**

Tibetans have lived at very high altitudes for thousands of years, and have a distinctive suite of physiological traits that enable them to tolerate environmental hypoxia. Expanding awareness and knowledge of the differences in hematology, hypoxia-associated genes, immune system of people living at different altitudes and from different ethnic groups may provide evidence for the prevention of mountain sickness.

**Method:**

Ninety-five Han people at mid-altitude, ninety-five Tibetan people at high-altitude and ninety-eight Han people at high-altitude were recruited. Red blood cell parameters, immune cells, the contents of cytokines, hypoxia-associated gene single nucleotide polymorphisms (SNPs) were measured.

**Results:**

The values of Hematocrit (HCT), Mean cell volume (MCV) and Mean cell hemoglobin (MCH) in red blood cell, immune cell CD19^+^ B cell number, the levels of cytokines Erb-B2 receptor tyrosine kinase 3 (ErbB3) and Tumor necrosis factor receptor II (TNF-RII) and the levels of hypoxia-associated factors Hypoxia inducible factor-1α (HIF-1α), Hypoxia inducible factor-2α (HIF-2α) and HIF prolyl 4-hydroxylase 2 (PHD2) were decreased, while the frequencies of SNPs in twenty-six Endothelial PAS domain protein 1 (EPAS1) and Egl-9 family hypoxia inducible factor 1 (EGLN1) were increased in Tibetan people at high-altitude compared with that of Han peoples at high-altitude. Furthermore, compared with mid-altitude individuals, high-altitude individuals showed lower blood cell parameters including Hemoglobin concentration (HGB), HCT, MCV and MCH, higher Mean cell hemoglobin concentration (MCHC), lower immune cells including CD19^+^ B cells, CD4^+^ T cells and CD4/CD8 ratio, higher immune cells containing CD8^+^ T cells and CD16/56NK cells, decreased Growth regulated oncogene alpha (GROa), Macrophage inflammatory protein 1 beta (MIP-1b), Interleukin-8 (IL-8), and increased Thrombomodulin, downregulated hypoxia-associated factors including HIF1α, HIF2α and PHD2, and higher frequency of EGLN1 rs2275279.

**Conclusions:**

These results indicated that biological adaption to hypoxia at high altitude might have been mediated by changes in immune cells, cytokines, and hypoxia-associated genes during the evolutionary history of Tibetan populations. Furthermore, different responses to high altitude were observed in different ethnic groups, which may provide a useful knowledge to improve the protection of high-altitude populations from mountain sickness.

## Introduction

Gansu Province in China is a place with various altitudes from about 900 m to 3800 m, having several ethnic groups including Tibetan and Han Chinese. However, people living at different altitudes for a long time have different physiological and immune functions affected by environmental factors. Environmental factors at high-altitude such as low oxygen partial pressure, UV exposure, very low temperature can affect the immune system and make it more susceptible to cancer, various infectious and autoimmune diseases. For example, at high-altitude >2500 m, several diseases including acute mountain sickness, headache, pulmonary edema, cerebral edema, skin cancer, hypertension, peptic ulcers, upper gastro-intestinal haemorrhage, gastric carcinoma, gastro-intestinal bleeding easily occur [1–3]. However, genetic adaptations have been evolved by high-altitude Tibetan populations to cope with such an extremely hypoxic environment [4–6].

The immune system is an interactive network of lymphoid organs, cells, humoral factors, and cytokines which work together to generate immune response against the invading and harmful stressors in the body. Factors at high-altitude have been known to stimulate inflammatory cytokines, activate adaptive immune cells such as T-cells, B-cells, NK cells which show a peculiar response to hypoxic stimuli [7, 8]. Studies have shown that an increase of lymphocyte numbers and the alterations in the release of cytokines produced by T lymphocyte present some signs of response to hypobaric stimulus [9]. Elevated levels of proinflammatory cytokines such as endothelin-1, IL-17a, IL-1β, IL-4, IL-6, IL-8, IL-10, hypoxia-inducible factor-1α and hypoxia-inducible factor-2α have been found in hypoxic subjects during ascent and sojourn at high-altitude [10–13]. However, it is not clear that whether more inflammatory cytokines and immune cells are relevant with generational people at high-altitude and with ethnic group.

For decades, studies have addressed the mechanisms of genetic adaptation to hypoxia that have allowed people such as Tibetans to more adaptively inhabit at high-altitude for many years [14–17]. Previous studies have reported that genetic variants at the EPAS1 and EGLN1 loci are under positive natural selection, associated with variation of hemoglobin concentration and HIF pathway [6, 12, 14]. These genes showed large allelic differences between Tibetans at high-altitude and Han Chinese at low-altitude [6, 14]. EPAS1, encoding hypoxia inducible factor-2α (HIF-2α) subunit, is involved in body response to hypoxia and plays an important role in regulating erythropoiesis [15–19]. EGLN1 encoding HIF prolyl 4-hydroxylase 2 (PHD2) is a major oxygen-dependent negative regulator of EPAS1 and HIF1α by hydroxylating them on specific proline residues [20–23]. Beall et al. found that EPAS1 (HIF-2α) was associated with low hemoglobin concentration in Tibetan at high-altitude [6]. Jeong et al, found a correlation between EPAS1 haplotype and low HGB in Tibetan, and raised the hypothesis that it was the oxygen-carrying portion of total HGB that drove the well-replicated association between EPAS1 SNPs and HGB [16]. Gnecchi-Ruscone et al, indicated that an adaptation to hypoxia was mediated not only by the few hard selective sweeps at genes involved in the erythropoietic cascade, but also by multiple subtle selective events at loci related to functional pathways involved in regulating angiogenesis [17]. Deng Wu et al, revealed that the positive selection of EPAS1 SNPs in Tibetans directly contributes to multiply their off-springs at high altitudes [24]. The low prevalence of chronic mountain sickness showed a correlation with a signal of selective sweep in the EPAS1 gene [5, 6] and unelevated HGB in Tibetan highlanders [25]. In additionally, EPAS1 polymorphisms showed associations with reduced susceptibility to high altitude polycythemia and high altitude pulmonary edema in Han populations [26, 27]. Taken together, it is suggested that EPAS1 gene variant may be a secondary adaptive mechanism useful to reduce risk of developing chronic mountain sickness in populations at high-altitude, but not the mechanism of increasing the fitness of Tibetan individuals. It has been reported that there is no difference in HIF-1A gene sequence between Andean native populations living at high altitude and people living at low altitude [28], but another study found that the GT14 allele was significantly more frequent in Sherpas living at high altitude (3750 meters) as compared with Japanese, native lowlanders. It can be seen that different ethnic groups have different genetic response to high altitude [29].

Therefore, the present study was to investigate hematology, cytokines, immune cells and single nucleotide polymorphisms (SNPs) of EPAS1, EGLN1 and HIF1A in mid-altitude Han, high-altitude Tibetan and Han, to reveal the adaption to hypoxia at different altitude and with different ethnic groups.

## Methods and materials

### Populations

95 Chinese Han individuals more than three generations from Lanzhou City, Gansu Province living at mid-altitude less than 1507 m, and 95 Tibetan and 98 Han individuals more than three generations living at high-altitude of 2900–4287 m from Luqu county locating at the junction of Gansu, Qinghai and Sichuan provinces and the east of Qinghai Tibet Plateau were recruited. All subjects resided in local region more than three generations and are healthy. Individuals with chronic obstructive pulmonary disease, pulmonary infection, asthma, polycythemia or congenital heart disease were excluded from the study. This study was approved by the ethics Committee of Dingxi People’s Hospital (NO: 2016A01). All the participants were informed the purpose and experimental procedures of the study.

### Blood cell parameters

Fasting venous blood sample from each individual was collected. Red blood cell (RBC) count, hemoglobin concentration (HGB), hematocrit (HCT), erythrocyte mean cell volume (MCV), mean cell hemoglobin (MCH), mean cell hemoglobin concentration (MCHC) in peripheral blood were measured by the SYSMEX XE-2100 (Sysmex, Kobe, Japan) Automatic Blood Cell Analyzer. The whole process from sample collection to clinical measurement was completed within 4 hours.

### Flow cytometry

Lymphocyte subsets and treg cells in peripheral blood were measured using flow cytometry analysis (FACScan, Becton Dickinson). Briefly, the peripheral blood cells were collected by centrifugation. Anti-CD3, anti-CD8, anti-CD19, anti-CD45, anti-CD4, anti-CD16, anti-CD56 antibodies were added into cell samples followed by an incubation in the dark at room temperature for 30 minutes. Anti-CD4, anti-CD25, anti-Foxp3 antibodies were added into cell samples then repeated the above steps. FACS hemolysis fluid was added for further incubation in the dark at room temperature for 15 minutes. The prepared samples were immediately tested by multiset (BD FACS Calibur flow cytometry).

### Antibody array assay

Serum samples from four Tibetan, four Han at high-altitude and four Han at mid-altitude were utilized for analysis of the circulating cytokine profile using an antibody array (Human Cytokine Antibody Array, RayBiotech, Norcross, GA, USA) simultaneously measuring 440 cytokines via a sandwich method. Briefly, serum was added into array pools and incubated overnight at 4 °C. After washing, 440 biotin-conjugated antibody mix was added into the array pools for 2 hours incubation at room temperature. Subsequently, Cy3-conjugated streptavidin was added into the array pools and incubated for 2 hours. Finally, the arrays were scanned by a microarray scanner (InnoScan 300, Innopsys, France) for the fluorescent signal visualization.

### ELISA

ELISA kits were performed using a larger sample size according to the manufacturer’s (Raybiotech, Inc.) instructions including 95 Tibetan people and 98 Han people at high-altitude, and 95 Han people at mid-altitude. Briefly, serum samples were added into the plate wells to incubate with capture antibody overnight at 4 °C. After washing, biotin-conjugated antibody was added into plate wells for 2 h incubation. And HRP-conjugated streptavidin was used to combine with biotin-conjugated antibody and catalyze following chromogenic substrate TMB. Finally, the catalytic reaction was stopped by sulfuric acid and the optical density was measured on an ELx800NBmicroplate reader (BioTek, Inc., Winooski, VT, USA).

### SNP selection and genotyping

Fifty-three tag SNPs of EPAS1, EGLN1 and HIF1 were analyzed in this study. Human genomic DNA was extracted using a standard method. The SNP genotyping work was performed using a custom-by-design 48-Plex SNPscan Kit (Cat#: G0104; Genesky Biotechnologies Inc., Shanghai, China). This kit was developed according to patented SNP genotyping technology by Genesky Biotechnologies Inc., which was based on double ligation and multiplex fluorescence PCR. Briefly, 100–200 ng of DNA sample was first denatured at 98 °C for 5 min in a 10 µL reaction containing 1x DNA lysis buffer and then mixed well with a 10 µL ligation premix composed of 2 µL 10x ligase buffer, 0.5 µL ligase, 1 µL probe mix, and 7.5 µL Milli-Q water. The ligation reaction was carried out in an ABI2720 thermal cycler. Two 48-plex fluorescence PCR reactions were performed for each ligation product. PCR reactions were prepared in a 20 µL mixture containing 1x PCR master mix, 1 µL primer mix set A or set B, and 1 µL ligation product. PCR products were separated and detected by capillary electrophoresis in an ABI3730XL sequencer. Raw data were analyzed according to the information obtained for the labeling dye color and fragment size of the allele-specific ligation-PCR product. Genotyping was conducted without any knowledge regarding the subject’s case or control status. For quality control, repeated analyses were accomplished to guarantee the genotyping quality by randomly choosing 4% of samples with high DNA quality.

### Statistical analysis

Statistical analysis was performed using the Bonferroni post hoc test of one-way ANOVA for any two groups of three groups by SPSS v20 (IBM Corp., Armonk, NY, USA) software. Differences were considered statistically significant for p < 0.05. SNPs were analyzed for Hardy-Weinberg Equilibrium Test.

## Results

### Differences of red blood cell parameters

**Fig. 1:**
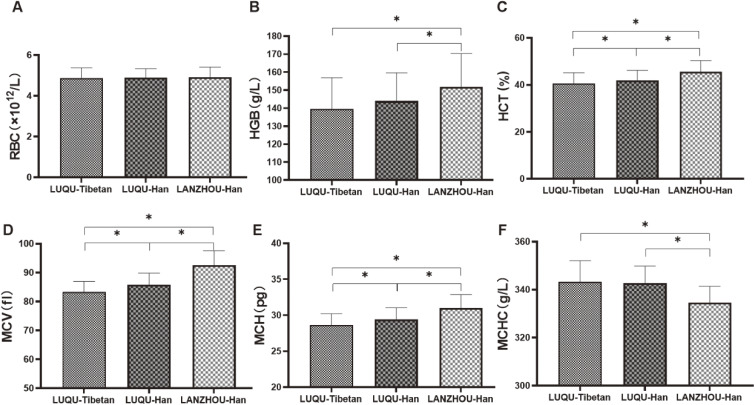
Detection of red blood cell parameters. The RBC, HGB, HCT, MCV, MCH and MCHC in blood from Tibetan population at high-altitude, Han people at high-altitude and Han individuals at mid-altitude were measured. LUQU-Tibetan: high-altitude Tibetan. LUQU-Han: high-altitude Han. LANZHOU-Han: mid-altitude Han. * p < 0.05.

As shown in Fig. 1, we found that values of HCT, MCV and MCH of Tibetan people at high-altitude were lower than those of Han people at high-altitude, and the values of HGB, HCT, MCV and MCH of people at high-altitude (both Tibetan and Han) were lower, while MCHC was higher than those of mid-altitude individuals. Taken together, it was revealed that RBC with an oxygen transport function might be not affected by altitude and ethnic group. HGB and MCHC showed a relation with altitude, where HGB was decreased and MCHC increased with the increase of altitude, but showed a non-relation with ethnic group. More important, HCT, MCV and MCH are not only related to ethnic group, but also related to altitude.

### Differences of immune cells

**Fig. 2:**
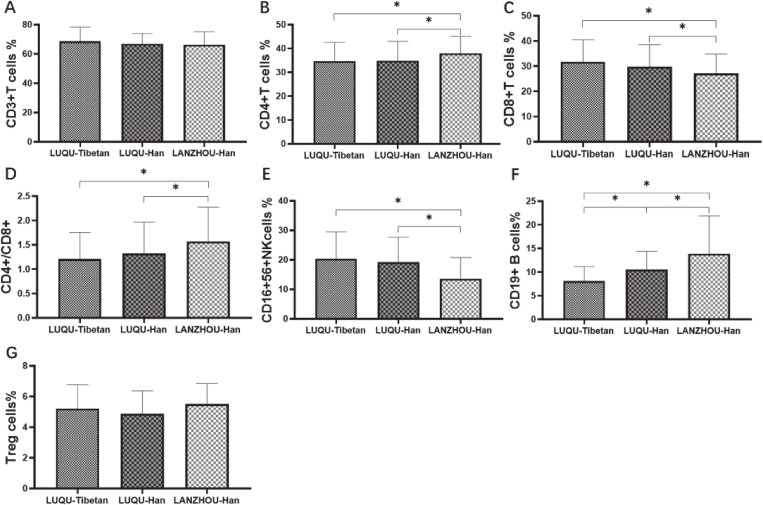
Detection of peripheral blood lymphocytes. CD19^+^ B cells, CD4^+^ T cells, CD8^+^ T cells, CD4/CD8 ratio, CD16/56NK cells, CD3^+^ T cells and CD4^+^ CD25^low^ Foxp3^+^ Treg cells from Tibetan population at high-altitude, Han people at high-altitude and Han individuals at mid-altitude were measured. LUQU-Tibetan: high-altitude Tibetan. LUQU-Han: high-altitude Han. LANZHOU-Han: mid-altitude Han. * p < 0.05.

In the detection of peripheral blood lymphocytes including CD19^+^ B cells, CD4^+^ T cells, CD8^+^ T cells, CD4/CD8 ratio, CD16/56NK cells and CD3^+^ T cells, as shown in Fig. 2, compared with Han people at high-altitude, only the CD19^+^ B cell number of Tibetan people at high-altitude were significantly lower, showing an association with ethnic group. While compared with Han individuals at mid-altitude, Tibetan and Han people at high-altitude had lower CD4^+^ T cells, CD19^+^ B cells and CD4/CD8 ratio, higher CD8^+^ T cells and CD16/56NK cells. Taken together, the immune cells including CD19^+^ B cells, CD4^+^ T cells, CD8^+^ T cells, CD4/CD8 ratio and CD16/56NK cells are closely related to altitude. While no significant differences in CD3^+^ T cells and CD4^+^ CD25^low^ Foxp3^+^ Treg cells were found among Tibetan population at high-altitude, Han people at high-altitude and Han individuals at mid-altitude.

### Differences of cytokines and hypoxic factors

**Fig. 3:**
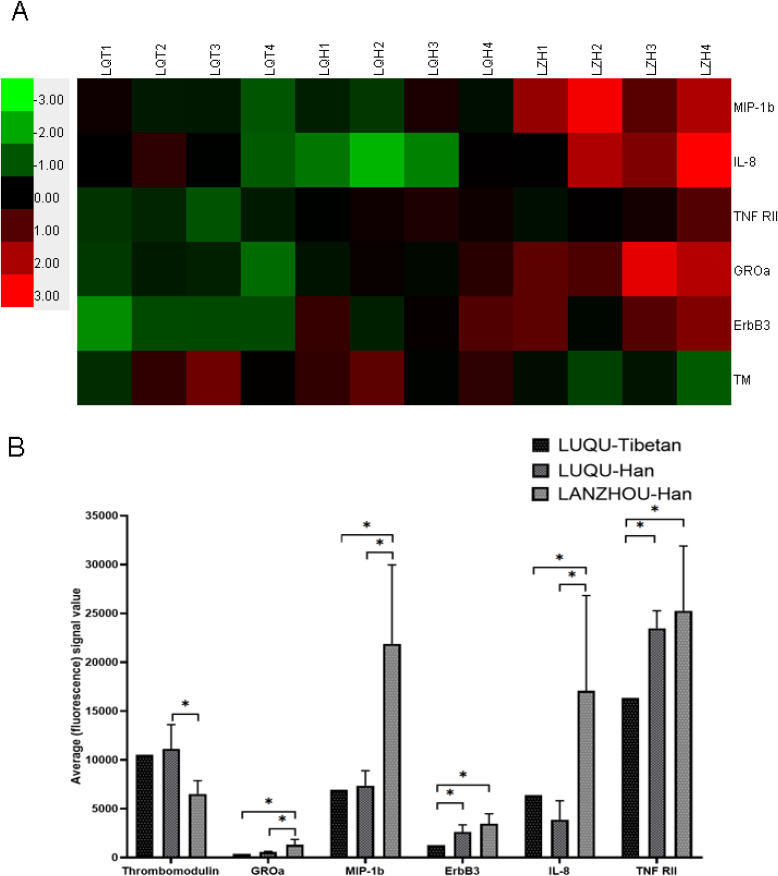
Detection of circulating cytokines. 440 cytokines were measured by antibody array, and GROa, MIP-1b, IL-8, Thrombomodulin (TM), ErbB3 and TNF-RII were significantly differential. A: Heatmap. Green indicates low levels of the proteins, black for median levels, and red for high levels. LQT: LUQU-Tibetan. LQH: LUQU-Han. LZH: LANZHOU-Han. B: Histogram. LUQU-Tibetan: high-altitude Tibetan. LUQU-Han: high-altitude Han. LANZHOU-Han: mid-altitude Han. * p < 0.05.

**Fig. 4:**
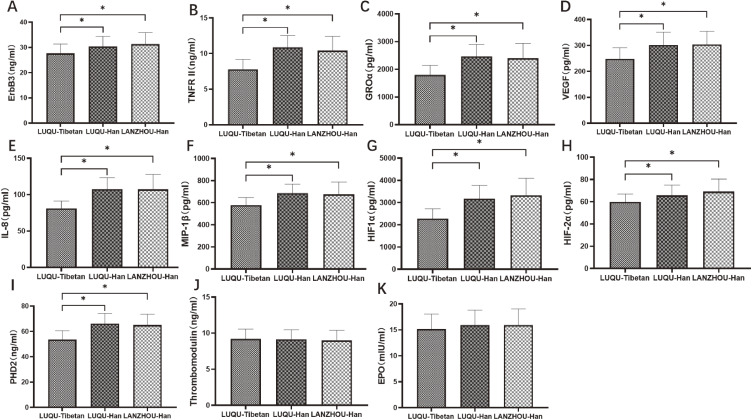
ELISA for cytokines and hypoxia-associated factors. GROa, MIP-1b, IL-8, Thrombomodulin, ErbB3 and TNF-RII from antibody array results was selected for validation and Hypoxia-associated factors HIF1α, HIF2α, PHD2 VEGF and EPO were measured by ELISA. LUQU-Tibetan: high-altitude Tibetan. LUQU-Han: high-altitude Han. LANZHOU-Han: mid-altitude Han. * p < 0.05.

In order to further investigate the immune mechanism of response to high altitude, circulating 440 cytokines including inflammatory factors, growth factors, chemokines and adhesion molecules were measured. As a result, compared with Han individuals at mid-altitude, contents of GROa, MIP-1b and IL-8 were decreased in Tibetan and Han people at high-altitude, while Thrombomodulin was increased, showing a relevance with altitude. ErbB3 and TNF-RII were decreased in Tibetan population at high-altitude when compared with Han people at high-altitude, suggesting a relation with ethnic group (Fig. 3A and B). Furthermore, the validation of TNF-RII and ErbB3 with larger sample size using ELISA for show consistent with the results of antibody array, and ELISA for hypoxia-associated factors showed HIF1α, HIF2α and PHD2 were decreased in Tibetan people at high-altitude compared with Han people at mid-altitude, and also were downregulated in Tibetan people at high-altitude compared with Han people at high-altitude (Fig. 4).

### Differences of tag SNPs in EGLN1, HIF-1α and EPAS1

**Fig. 5:**
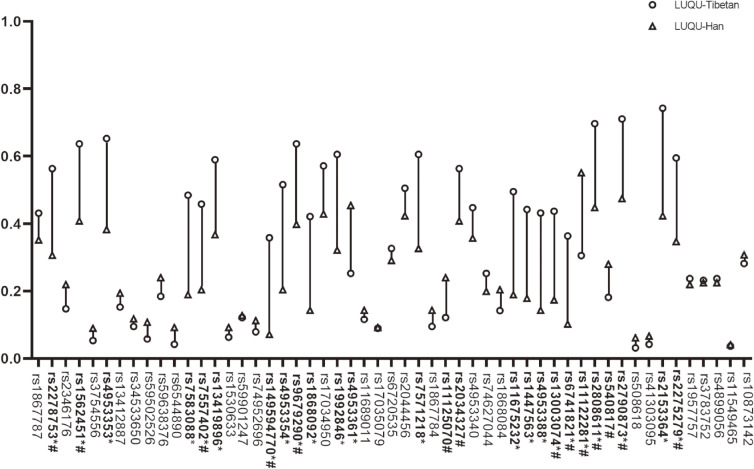
The SNP analysis of EGLN1, EPAS1 and HIF1α between Tibetan and Han living in high-altitude. SNPs marked in bold were significantly differential with p < 0.05. * Bofferoni adjusted p < 0.05. # Newly discovered SNPs.

**Fig. 6:**
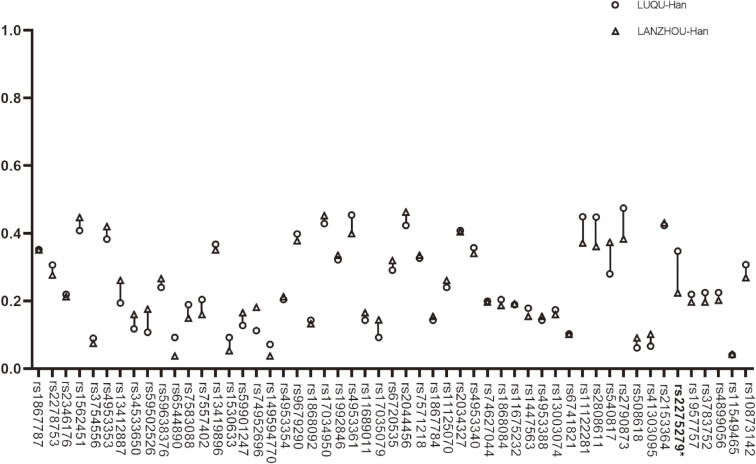
The SNP analysis of EGLN1, EPAS1 and HIF1α between mid-altitude Han and high-altitude Han. EGLN1 rs2275279 marked in bold was significantly differential with p < 0.05.

Fifty-three tag SNPs of EGLN1, HIF-1α and EPAS1 genes were detected and the information of SNP sites was shown in Table 1. The results showed that between Tibetan people at high-altitude and Han people at high-altitude there were differences in the SNPs of EGLN1 and EPAS1 including rs2278753, rs1562451, rs4553353, rs7583088, rs7557402, rs13419896, rs149594770, rs4953354, rs9679290, rs1868092, rs1992846, rs4953361, rs7571218, rs11125070, rs2034327, rs11675232, rs1447563, rs4953388, rs13003074, rs6741821, rs11122281, rs2808611, rs540817, rs2790873, rs2153364 and rs2275279 (Fig. 5). Furthermore, among these SNPs, rs2278753, rs1562451, rs7557402, rs149594770, rs9679290, rs11125070, rs2034327, rs13003074, rs6741821, rs11122281, rs2808611, rs540817, rs2790873 and rs2275279 were firstly found in this study. However, without regard to national differences, we found that there was significant difference only in SNP rs2275279 of EGLN1 gene between mid-altitude and high-altitude Han people (Fig. 6).

**Table 1:** The information of fifty-three tag SNPs of EGLN1, HIF-1α and EPAS1 genes

**No.**	**rs_num**	**Chr**	**Position_37**	**SNPs**	**GeneName**
1	rs1867787	2	46525391	G/C	EPAS1
2	rs2278753	2	46583593	G/A	EPAS1
3	rs2346176	2	46593299	T/C	EPAS1
4	rs1562451	2	46579730	T/C	EPAS1
5	rs3754556	2	46595490	G/A	EPAS1
6	rs4953353	2	46567276	T/G	EPAS1
7	rs13412887	2	46589487	G/C	EPAS1
8	rs34533650	2	46572449	G/A	EPAS1
9	rs59502526	2	46573395	T/C	EPAS1
10	rs59638376	2	46560090	G/A	EPAS1
11	rs6544890	2	46586114	T/A	EPAS1
12	rs7583088	2	46603165	G/A	EPAS1
13	rs7557402	2	46603671	G/C	EPAS1
14	rs13419896	2	46556345	G/A	EPAS1
15	rs1530633	2	46590456	G/A	EPAS1
16	rs59901247	2	46609572	C/A	EPAS1
17	rs74952696	2	46595256	G/A	EPAS1
18	rs149594770	2	46552202	T/A	EPAS1
19	rs4953354	2	46575388	G/A	EPAS1
20	rs9679290	2	46557644	G/C	EPAS1
21	rs1868092	2	46614202	G/A	EPAS1
22	rs17034950	2	46538794	G/A	EPAS1
23	rs1992846	2	46597581	T/C	EPAS1
24	rs4953361	2	46598568	G/A	EPAS1
25	rs11689011	2	46541176	T/C	EPAS1
26	rs17035079	2	46596951	T/A	EPAS1
27	rs6720535	2	46547494	G/A	EPAS1
28	rs2044456	2	46546316	G/A	EPAS1
29	rs7571218	2	46605659	G/A	EPAS1
30	rs1867784	2	46534220	T/C	EPAS1
31	rs11125070	2	46552926	T/A	EPAS1
32	rs2034327	2	46549040	G/C	EPAS1
33	rs4953340	2	46548064	G/C	EPAS1
34	rs74627044	2	46546990	G/A	EPAS1
35	rs1868084	2	46570708	G/C	EPAS1
36	rs11675232	2	46597870	T/C	EPAS1
37	rs1447563	2	46637364	C/A	EPAS1
38	rs4953388	2	46713201	G/A	EPAS1
39	rs13003074	2	46671420	T/A	EPAS1
40	rs6741821	2	46723365	G/C	EPAS1
41	rs11122281	1	231519842	G/A	EGLN1
42	rs2808611	1	231548480	G/A	EGLN1
43	rs540817	1	231541686	T/C	EGLN1
44	rs2790873	1	231539685	G/A	EGLN1
45	rs508618	1	231532312	G/A	EGLN1
46	rs41303095	1	231500238	G/A	EGLN1
47	rs2153364	1	231560220	G/A	EGLN1
48	rs2275279	1	231727094	T/A	EGLN1
49	rs1957757	14	62196948	T/C	HIF1A
50	rs3783752	14	62185692	G/A	HIF1A
51	rs4899056	14	62189531	T/C	HIF1A
52	rs11549465	14	62207557	T/C	HIF1A
53	rs10873142	14	62203462	T/C	HIF1A

## Discussion

It is cold and hypoxic at high altitude and hypobaric hypoxia is a problem in individuals not genetically adapted to high altitude at high-altitudes, resulting in reduced blood arterial oxygen saturation, hypoxemic blood. In the present study, we found that RBC was not affected by altitude and ethnic group, HGB and MCHC only showed a relation with altitude, while HCT, MCV and MCH were not only related to ethnic group, but also related to altitude.

**Fig. 7:**
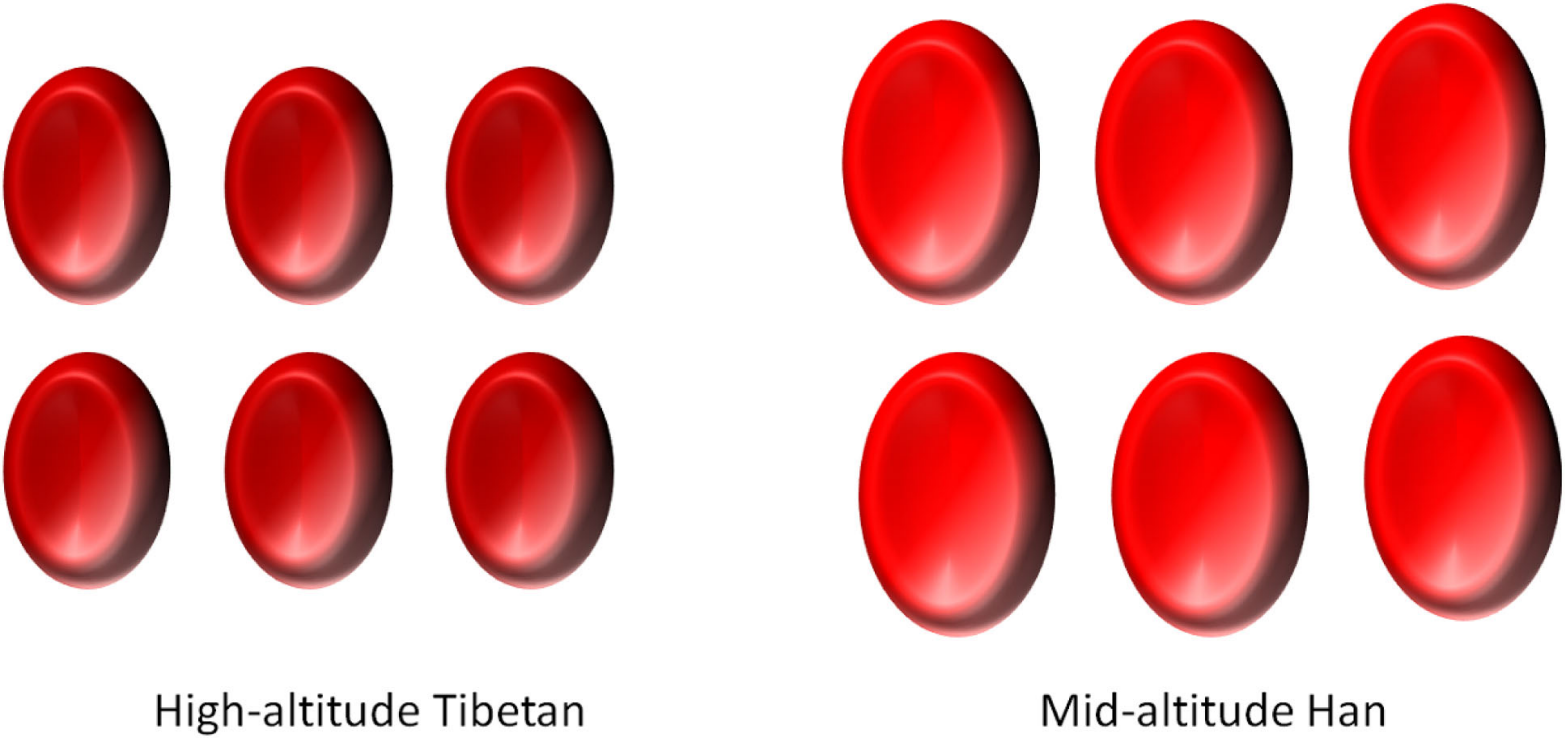
Pattern of mature red blood cell morphology in Tibetan and Han ethnic group. High-altitude Tibetan on the left and mid-altitude Han on the right.

The deformability of RBC plays a crucial role in transporting and exchanging oxygen in the blood, and in removing respiratory byproducts in organisms. Christopher et al. reported that increases in red blood cell production did not happen after 4 weeks of exposition to hypobaric hypoxia [30], suggesting it is possible that deformability of RBC rather than RBC count is responsible for hematological responses to hypoxia for long-term residence in high altitude. Our data show that MCV of Tibetan people at high-altitude is smaller and the MCHC is higher, forming a concentrated red blood cell (Fig. 7). Furthermore, previous studies revealed that the people permanently living at an altitude possess heritable adaptations to the hypoxic environment, as indicated by lower HGB, HCT, MCV and MCH, higher oxygen saturation of blood [31–35]. HCT in Tibetans was lower than that in Han, showing a relation with ethnic group [36], which are consistent with our present findings.

In addition, high altitude hypoxia is known to induce the immune response and affect the differentiation and functions of immune cells, such as lymphocytes including T cells, B cells and natural killer (NK) cells [37–39]. In the present study, lower CD4^+^ T cells, CD19^+^ B cells and CD4/CD8 ratio, higher CD8^+^ T cells and CD16/56NK cells were found in peripheral blood of Tibetan and Han people at high-altitude. It is known that exposure to high altitude hypoxia may influence T cell immunity [40]. Recent observations indicated that the exposure to hypoxia caused a decrease of circulating CD4^+^ T cells [9, 39]. Although evidences showed a non-significant change of CD8^+^ T cells in high altitude [9, 41], Caldwell et al. [7] and our present study found that low oxygen favored more CD8^+^ T cells development. CD4/CD8 ratio is an important marker of immune health, and CD4/CD8 ratio <1 is associated with activation of inflammation. Decreased CD4/CD8 ratio caused by lower CD4^+^ T cells and higher CD8^+^ T cells may activate the inflammatory response of people at high-altitude. B cells have crucial roles in cytokine production, regulation of lymphoid organogenesis and effector T-cell differentiation. CD19 serves as a positive B-cell response regulator. Although hemodialysis patients with lower count of CD19^+^ B cells have higher risk of mortality [42], there had been fewer researches on the relationship of CD19^+^ B cells with hypoxia. In the present study, lower CD19^+^ B cells existing in altitude population might impair their immune system. NK-cell is an important mediator in immunity, and is enhanced in humans exposed to high altitude [43]. Mishra et al. [44] revealed that the percentage of NK cells (CD16/56) was upregulated in hypoxia exposure, consistent with our finding, suggesting NK cells can adapt to the hypoxic environment. We also analyzed Treg cells from different populations, and there was no significant difference.

In addition, immune cells also release cytokines as critical mediators to regulate the immune response, and participate in the communication of signals in both innate and adaptive immunity. Particularly, cytokines released by CD4^+^ T cells at the onset of an immune response are essential for pathological or physiological processes. Therefore, we further revealed the levels of cytokines in plateau population. As a result, antibody array assay showed compared with mid-altitude individuals, GROa, MIP-1b and IL-8 were decreased in people at high-altitude, while Thrombomodulin was increased, showing an association with altitude. Moreover, ERBB3 and TNF-RII were decreased in Tibetan population at high-altitude when compared with Han people at high-altitude, suggesting a relationship with ethnic group. We verified the differential factors screened by microarray, and at the same time, we determined the key factors of hypoxia pathway, HIF1α, HIF2α, PHD2, VEGF and EPO by ELISA, Furthermore, ELISA for the validation of TNF-RII and ErbB3 with larger sample size show the consistency with the results of antibody array, and ELISA for hypoxia-associated factors HIF1α, HIF2α and PHD2 showed that they were not only related to ethnic group, but also related to altitude.

GROa, MIP-1b and IL-8 are chemokines in charge of cell recruitment and activation under homeostatic and inflammatory conditions. GROa (CXCL1) and IL-8 are members of the CXC chemokine family. GROa is essential for neutrophil migration, expression of proinflammatory mediators. Previous researches focused on the relationship of hypoxia and CXCL1 in tumor, and showed the effect of hypoxia on the expression of CXCL1 depended on the type of cancer. However, fewer studies revealed CXCL1 expression in high altitude population. IL-8, a chemotactic cytokine for T lymphocytes and neutrophils, is known to precede acute phase response to an inflammatory stimulus. Many studies reported hypoxia caused IL-8 secretion in cancers and high-altitude diseases [45–48]. The CXC chemokine macrophage inflammatory protein 1β (MIP-1b) is a chemoattractant preferentially activating and migrating CD4^+^ T lymphocytes [49], so we speculate decreased MIP-1b in high-altitude population may be a cause of lower CD4^+^ T cells in this study. In addition, MIP-1b enhanced the survival effect of neutrophils under hypoxia [50]. Our present study showed GROa, MIP-1b and IL-8 were decreased in people at high-altitude. Roach et al. [51] demonstrated elevated levels of anti-inflammatory factors in individuals resistant to altitude illness. Therefore, it was suggested long-term residence in high altitude may inhibit the inflammatory response for the adaptation to hypoxia via the downregulation of inflammatory factors. ERBBs are associated with Th1, Th2, Th17, and Treg cellular immune markers, and TNF-RII is a transmembrane receptor that is linked to immune modulation, suggesting there may be a differential immune system in different ethnic group.

**Fig. 8:**
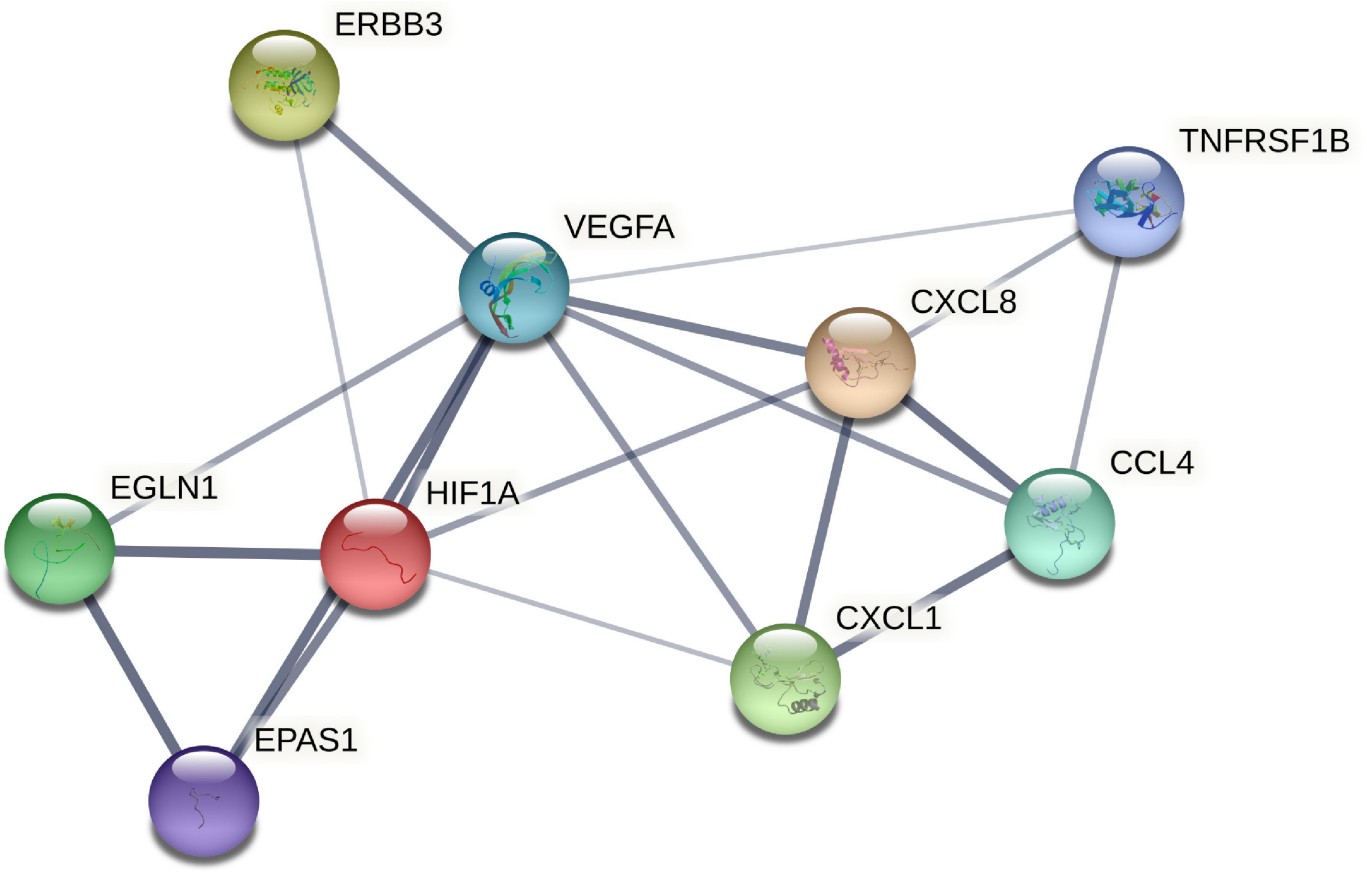
Protein-protein interaction analysis. Lines between two proteins denotes biological functional correlation and line thickness denotes the strength of the data support. The cytokines of MIP-1-beta(CCL4), GROa(CXCL1), IL-8(CXCL8), PHD2(EGLN1), HIF-2α(EPAS1); ErbB3, TNF-RII; HIF1α and VEGFA have strong correlation.

HIF, an endogenous hypoxia marker, functions as master regulators of oxygen homeostasis facilitating an adaptive response to hypoxia, and plays crucial roles in the regulation of development, differentiation, and functions of immune cells [52]. Hypoxia inducible factor-1α (HIF-1α) is known to regulate the expression of various chemokines, including IL-8, GROa and MIP-1b [45, 53, 54]. Also as analyzed by protein-protein interaction (Fig. 8), HIF-1α, VEGF and IL-8 locating in the central position interact with other proteins, suggesting HIF-1α may facilitate an adaptive response to hypoxia via regulating VEGF and IL-8 which further acts on other cytokines including GROa, MIP-1b, TNF-RII. In addition, HIF-1α and HIF-2α have been implicated in the pathogenesis of pulmonary hypertension [55], which may represent an important marker for determination of high-altitude pulmonary edema susceptibility [56]. Prolyl hydroxylase domain protein 2 (PHD2) is a key oxygen sensor, setting low level of HIF-α [57]. Therefore, it was supposed that decreased PHD2 might facilitate high-altitude individuals an adaptive response to hypoxia via downregulating HIF-1α and HIF-2α which further affected chemokines IL-8, GROa and MIP-1b and immune cells, which needs further research. Some microarray results are inconsistent with ELISA, which may be explained by the small sample size of microarray. Moreover, the gene polymorphisms of these hypoxia-associated factors also involve into high-altitude adaptation. In the present study, the SNPs of EPAS1 and EGLN1 including rs2278753, rs1562451, rs7557402, rs149594770, rs9679290, rs11125070, rs2034327, rs13003074, rs6741821, rs11122281, rs2808611, rs540817, rs2790873, rs2275279 were firstly found associated with ethnic group in high altitude. It might be the facts the SNPs variants in hypoxia pathway genes that are present at high frequency only in high altitude populations appear to decrease HIF activity and blunt the erythropoietic response to hypoxia. We analyzed the SNPs among Han people living at high-altitude more than three generations, Han people living at mid-altitude and indigenous Tibetans in high-altitude, these SNPs presented higher frequencies in Tibetan than Han groups. It is suggested that Han people short-term living at high-altitude have not evolved genetic adaptation to hypoxia, and higher frequencies of SNPs in Tibetan may endow them a plateau adaptation. At the same time, many SNPs of EPAS1 and EGLN1 genes in Gansu Tibetan are consistent with some loci in Tibetans from Qinghai-Tibet Plateau, suggesting that they may originate from the same ancestor. Evidence showed that PHD^2D4E; C127S^ variant suppressed monocyte function [58], and our results exhibited that immune cell CD19^+^ B cell, immune factors ERBBs and TNF-RII, hypoxia-associated factors HIF1α, HIF2α and PHD2 were different between Tibetan and Han in high altitude, suggesting genetic variants of the hypoxia-associated factors may cause different immune system in different ethnic groups. Furthermore, it was firstly found that for the SNP rs2275279 of EGLN1 gene, the A allele had a high frequency in high-altitude individuals, indicating A allele of rs2275279 may play a role in the adaption response to hypoxia. At the same time, many SNPs of EPAS1 and EGLN1 genes of Tibetan in Gansu province studied in this project are consistent with some loci reported by Tibetan in Qinghai-Tibet plateau, and it is speculated that they may have originated from the same ancestor. Therefore, the people living at high altitude are different from those rushing to high altitude. Genes from hypoxia pathway including EPAS1 and EGLN1 play an important role in response to the hypoxic stress during adaptation to low oxygen conditions [25]. In fact, genetic variants arise randomly according to mutational processes or enter the gene pool of populations due to gene flow from other populations [59]. Several millennia of exposition to hypoxia thus did not cause genetic changes, but only favored the individuals presenting them. These gene SNPs hereditarily in Tibetan may give them long-term adaptation to hypoxia, contribute to morphological deformation of red blood cells, down-regulation of related immune cells and cytokines.

In conclusion, our present study showed red blood cell parameters, immune cell, cytokines, SNPs of EPAS1 and EGLN1 were different between Tibetan and Han in different altitude, and these results indicated genetic variants of hypoxia-associated factors between different ethnic groups might cause different immune system via immune cells and immune factors for their adaption to hypoxia. However, these conjectures need further researches.

## Declarations

### Availability of data and materials

Data available on request from authors.
